# Small-scale variation in a pristine montane cloud forest: evidence on high soil fungal diversity and biogeochemical heterogeneity

**DOI:** 10.7717/peerj.11956

**Published:** 2021-08-11

**Authors:** Patricia Velez, Yunuen Tapia-Torres, Felipe García-Oliva, Jaime Gasca-Pineda

**Affiliations:** 1 Instituto de Biología, Universidad Nacional Autónoma de México, Mexico City, Mexico; 2 Escuela Nacional de Estudios Superiores Unidad Morelia, Universidad Nacional Autónoma de México, Morelia, Michoacán, Mexico; 3 Instituto de Investigaciones en Ecosistemas y Sustentabilidad, Morelia, Universidad Nacional Autónoma de México, Morelia, Michoacán, Mexico; 4 UBIPRO, Facultad de Estudios Superiores Iztacala, Universidad Nacional Autónoma de México, Estado de México, Mexico

**Keywords:** Ascomycota, Basidiomycota, C:N:P stoichiometry, Myco-diversity, Mortierella, Spatial heterogeneity

## Abstract

Montane cloud forests are fragile biodiversity hotspots. To attain their conservation, disentangling diversity patterns at all levels of ecosystem organization is mandatory. Biotic communities are regularly structured by environmental factors even at small spatial scales. However, studies at this scale have received less attention with respect to larger macroscale explorations, hampering the robust view of ecosystem functioning. In this sense, fungal small-scale processes remain poorly understood in montane cloud forests, despite their relevance. Herein, we analyzed soil fungal diversity and ecological patterns at the small-scale (within a 10 m triangular transect) in a pristine montane cloud forest of Mexico, using ITS rRNA gene amplicon Illumina sequencing and biogeochemical profiling. We detected a taxonomically and functionally diverse fungal community, dominated by few taxa and a large majority of rare species (81%). Undefined saprotrophs represented the most abundant trophic guild. Moreover, soil biogeochemical data showed an environmentally heterogeneous setting with patchy clustering, where enzymatic activities suggest distinctive small-scale soil patterns. Our results revealed that in this system, deterministic processes largely drive the assemblage of fungal communities at the small-scale, through multifactorial environmental filtering.

## Introduction

Montane cloud forest (MCF) makes up barely 2.5% of the total area of the tropical forests globally. It has a patchy distribution, restricted to ravines along mountain slopes from 600 to 2,000 m above sea level (Rzedowski, 1996), and is characterized by the persistence of clouds and mist (Hamilton, Juvik & Scatena, 1995). In spite of its restricted distribution, this ecosystem harbors a remarkably vast biodiversity, being home to a number of threatened and endemic species (Bubb et al., 2004). In the megadiverse Mexico, MCF (*sensu* Rzedowski, 1978; Rzedowski, 1996) is recognized as the terrestrial ecosystem with the highest diversity per unit area, holding around 10% of the overall estimated flora diversity, more than 60% fern species, and around 30% of endemic species (Rzedowski, 1991; Tejero-Díez, Torres-Díaz & Gual-Díaz, 2014) in the country.

Worldwide the extent of threatened ecosystems is alarmingly constantly increasing, yet underestimated (Morrison et al., 2020). So, ecosystem monitoring is critical for identifying urgency of management responses and assessing the efficacy of management interventions (Lindenmayer et al., 2020). The World Wildlife Fund catalogues 86% of the MCFs identified by the United Nations Environment Program within the Global 200 Priority Forest Ecoregions for Nature. This ecosystem is one of the most threatened biomes, covering less than 1% of northern Mesoamerica (Aldrich et al., 2000; Challenger, 1998; Luna-Vega et al., 2006). In Mexico, MCF has a fragmented distribution that is framed by deforestation, changes in land use (Dirzo, 1992; Luna, Almeida-Leñero & Llorente-Bousquets, 1989; Luna, Ocegueda & Alcántara, 1994; Pineda et al., 2005), and is projected to reduce by 68% in 2080 as a result of climate change (Rojas-Soto, Sosa & Ornelas, 2012). Hence, to attain its conservation, disentangling diversity patterns at all levels of ecosystem organization is mandatory.

Inferences made in spatial ecology depend strongly on the scale of observation, as patterns that are evident at certain scale may be absent at other (*e.g*. Catalán, Valdivia & Scrosati, 2020; Coleman, 2002; Davies et al., 2005; Wiens, 1989). Therefore, a better understanding of ecosystems depends on an integrative approach at different scales (Oda et al., 2019), from intra-individual to ecosystem level. Since early investigations, heterogeneity in natural systems has been repeatedly documented at small scales such as a single tree level (Branco, Bruns & Singleton, 2013), 36 m^2^ (Štursová et al., 2016), and even in less than a meter (*e.g*. Downes & Beckwith, 1951; Frankland, Ovington & Macrae, 1963; Snaydon, 1962). These small-scale variations (*e.g*. in biogeochemistry and soil characteristics) have been demonstrated to play a fundamental role in shaping the species distributions (Cartwright, 2019), impacting important ecosystem level processes (*e.g*. nutrients dynamics, Loiselle et al., 2016). However, in MCFs studies at this scale have received less attention with respect to larger macroscale explorations (*e.g*. elevation gradients revised in Looby & Martin (2020)), hampering the robust view of ecosystem functioning as small-scale processes may be masked by larger scale features (Domínguez-Yescas et al., 2020; Krashevska, Maraun & Scheu, 2010; Stoyan et al., 2000).

Soils are among the vastest (Whitman, Coleman & Wiebe, 1998) and most biodiverse habitats on Earth for microbial life (Quince, Curtis & Sloan, 2008), promoting ecosystem multifunctionality (Delgado-Baquerizo et al., 2020). Nevertheless, we still have a rudimentary view of this complex system as a result of three major limitations. First, it has been estimated that less than 1% of microbial diversity is detectable in the laboratory by culture-dependent techniques (Torsvik & Øvreås, 2002); hence, our understanding of microbial diversity is biased by methodological limitations when culturing microorganisms. Second, the relationship between taxonomic and functional microbial diversity remains largely unknown (papers targeting fungi are still rare, since most of the studies focus on prokaryotes, *e.g*. Likhitrattanapisal et al., 2021), hampering the interpretation of nutrient transformations (Nannipieri et al., 2003) and spatial scale allocation of resources (*e.g*. mycorrhizal networks *e.g*. Püschel et al., 2020). Third, although gross trends in microbial communities may be approached from large-scale surveys (Adamczyk et al., 2019; Castro et al., 2010; Kuramae et al., 2012; Pinto-Figueroa et al., 2019; Seppey et al., 2020), these strategies are not always best suited to examine fine-scale factors that drive and maintain microbial diversity, such as environmental patchiness and the occurrence of microhabitats (Holden, Ritz & Young, 2011; Krashevska, Maraun & Scheu, 2010; Vos et al., 2013).

Fungi occur in soil as an abundant and diverse element, principally near organic material such as roots and root exudates (Blackwell, 2011). With more than 3,300 soil-borne species (Gams, 2007), these largely microscopic osmotrophs play key roles in maintaining ecosystem functionality (*e.g*. Baldrian et al., 2011; Osono, 2006), being fundamental for the maintenance of energy flow within the soil food web (Delgado-Baquerizo et al., 2020). Even though large-scale fungal community-level studies in soil are increasing (Averill et al., 2019; Bui et al., 2020; Tedersoo et al., 2014; Xue et al., 2018), only a few have been conducted at a local scale (*e.g*. Bahram, Peay & Tedersoo, 2015), and scarcer at a small-scale (*e.g*. Branco, Bruns & Singleton, 2013; Peay, Bidartondo & Arnold, 2010; Štursová et al., 2016). Particularly, fungal diversity in MCFs has been grossly investigated at the large-scale across elevation gradients, suggesting community composition shifts epitomized by the decrease of *Glomeromycota* (forming arbuscular mycorrhiza) with elevation, yet there is still scarce information on this matter (Bueno et al., 2021; Looby, Maltz & Treseder, 2016; Toussaint et al., 2020). Currently, at large, fungal records in Neotropical MCF, account for 2,962 species, with the *Basidiomycota* as a dominant component (del Olmo-Ruiz et al., 2017). Though, this estimation might be prominently biased by insufficient information on uncultivable taxa only present in natural conditions.

The generation of robust baseline data targeting the small-scale evaluation of key soil taxa, such as fungi, is crucial to understand the current status of MCFs and to forecast future trends, promoting their management and conservation. So, the aim of this work was to explore soil fungal taxonomic and functional diversity, community structure (species composition), and niche characteristics (understood as the position of a species in relation to local environmental variables necessary for its persistence, and its ecological role, *sensu* Lu et al., 2020) across a 10 × 10 × 10 triangular transect in a pristine MCF of Mexico, through the implementation of high-throughput sequencing of PCR products amplified from environmental DNA samples and biogeochemical profiling of soil samples. We hypothesize that the herein investigated pristine MCF harbors a high fungal diversity in relation to the reported numbers for Mexico, and that fungal community structure is shaped by deterministic processes that are linked to the availability of soil nutrients at the small-scale.

## Materials and Methods

### Study area

Fieldwork was conducted in the MCF of El Relámpago Mount (17° 35′ 30.4″ N, 96° 23′ 57.1″ W), municipality of Santiago Comaltepec, in the Northern Oaxaca Mountains, Mexico. This territory possesses remarkably high species diversity (including endemisms and species in protected status), and it is conserved by active social practices (Ponce-Reyes et al., 2012; Toledo-Aceves et al., 2011). Currently, this MCF is recognized for its good conservation status (del Mar Delgado-Serrano, Escalante & Basurto, 2015), being identified as an extreme priority site for biodiversity conservation in Mexico (CONABIO-CONANP-TNC-Pronatura-FCF/UANL, 2007; Ponce-Reyes et al., 2012).

In this area, MCF grows from 750 to 2,200 m above sea level, neighboring further down with tropical forests and higher up there are pine-oak forests. Vegetation includes *Oreomunnea mexicana* (Standl.) J.-F. Leroy, epiphytes, ferns and lycophytes; and the principal soil type is acidic humic Acrisol (Rzedowski, 1996; Sundue, 2017). It has a temperate-humid climate with rainy season in summer, mean annual temperature of 15 °C, and an average annual precipitation of 1,500 mm.

### Samples collection

Three sampling sites were established along an equilateral 10 m-triangular transect (spatial sampling method that increases the chance of achieving a high interstrata variance; Bąk, 2014) at 2,219 masl: (A) 17° 35′ 0.36″ N, 96° 23′ 36.6″ W (adjacent an individual of *O. mexicana*), (B) 17° 35′ 18.4″ N, 96° 23′ 57.7″ W (proximate to an arborescent fern *Alsophila salvinii* Hook), (C) and 17° 34′ 51.83″ N, 96° 24′ 5.17″ W (near a fallen decaying tree). Subsequently, in each vertex, a 1 m-equilateral sub-transect was traced. In total, nine soil cores (three subsamples per site) were collected in the first 25 cm layer (excluding litter) using a 10 cm diameter corer, in each vertex of the sub-transects. Soil samples were collected in sterile hermetic plastic bags that were immediately transported to the laboratory (within the next 24 h after collection) for processing in a cooler (approx. at 4 °C in absolute dark), where they were preserved at −80 °C until processing (within the next 48 h after collection). Samplings were conducted under the field research permit issued by Secretaría de medio Ambiente y Recursos Naturales, Subsecretaría de Gestión para la Protección Ambiental, Dirección General de Gestión Forestal y de Suelos (SGPA/DGGFS/712/2955/17), of the Mexican government.

### Soil DNA isolation, amplification and sequencing

Direct extraction of total DNA was performed for each soil subsample (0.25 g), using DNeasy PowerSoil kit (Qiagen, Hilden, Germany) according to the instructions provided by the manufacturer. The DNA samples were quantified using Qubit^®^ 2.0 Fluorometer (Invitrogen by Life Technologies, Carlsbad, CA, United States), and sequenced using Illumina MiSeq paired-end format (2 × 300) by the commercial service provided by the Genomic Services Laboratory (LANGEBIO, Irapuato, Guanajuato, Mexico; where the steps of amplification, library preparation, and sequencing were done), targeting the ITS1 region of the ribosomal RNA gene cluster (using primers set ITS1-F and ITS2; Gardes & Bruns, 1993; White et al., 1990), yielding to around 25,000 reads per subsample (available under the GenBank accession no. PRJNA634568). The ITS1 region was selected as a fungal barcode, since it has been repeatedly reported to outperform the ITS2 in terms of richness and taxonomic coverage (Mbareche et al., 2020), and provides a number of advantages such as its higher variability in relation to the ITS2 (Monard, Gantner & Stenlid, 2013; Wang et al., 2015). Additionally, the higher amount of available ITS1 sequences in databases (as this region is broadly used to assess fungal diversity), allows a better resolution when delimiting fungal groups (Mbareche et al., 2020).

### Fungal taxonomical assignment

The ITS Illumina raw reads were processed using the fungal ITS-specific workflow of the R v3.5.1 (R Development Core Team, 2018) package dada2 v1.13.1 (Callahan et al., 2016). Briefly, cutadapt was used to remove primer and adapter contamination from the raw reads (Martin, 2011). Then, to determine amplicon sequence variants (ASVs), we excluded reads with ambiguous bases (maxN = 0), and each read was required to have <1 expected errors based on their quality scores (maxEE = c (1,1), truncQ = 3). Therefore, ASVs were inferred for forward and reverse reads for each subsample using the run-specific error rates. To assemble paired-end reads, we considered a minimum of 50 pb of overlap and excluded reads with mismatches in the overlapping region. Chimeras were removed using the consensus method of “removeBimeraDenovo” implemented in dada2, and singletons were excluded of the analysis. The taxonomic assignment was performed using the DECIPHER v2.14.0 Bioconductor package against the eukaryotic UNITE_v2019_July2019 database (Nilsson et al., 2019) using the trained classifier available on DECIPHER website (http://www2.decipher.codes/Downloads.html). The ITS dataset was filtered for non-fungal reads. To avoid diversity overestimation due to over-split of ASVs with taxonomic assignment higher to genus (for example, *Basidiomycota* sp.), we re-clustered these assignments using Cd-Hit (Li & Godzik, 2006) with a threshold of 97%. Sampling intensity was assessed using rarefaction and extrapolation curves with the R package iNEXT v 2.0.20 (Hsieh, Ma & Anne, 2020).

### Diversity analyses

Graphics of the relative abundance and intersection plots were created using the R packages ggplot2 (Wickham, 2016) and UpSetR (Conway, Lex & Gehlenborg, 2017). Alpha-diversity was evaluated using the Shannon diversity index and evenness (Shannon, 1948). To characterize the fungal taxonomic composition among subsamples per site, we estimated Bray–Curtis (abundance-based; 1957), and Sorensen dissimilarity (presence-absence; Sørensen, 1948) matrices. We used constrained correspondence analysis (CCA hereafter) to represent the differences among subsamples and sites using a Hellinger-transformed abundance table (Legendre & Gallagher, 2001), excluding the unclassified sequences. To evaluate the effect of the sampling sites in the taxonomic composition, we conducted a Permutational Multivariate Analysis of Variance (PERMANOVA) using the Sorensen and Bray-Curtis dissimilarity matrices, and the sites as grouping factor; the significance was assessed with 10,000 permutations. All analyses were performed using the R package “vegan” v2.5-6 (Oksanen et al., 2019).

To examine the role of stochastic factors in community beta-diversity, we implemented the Raup–Crick metric (Raup & Crick, 1979) with the modification proposed by Stegen et al. (2012). This approach estimates probability of the number of shared species between two communities through randomizations of the sample pool. Then, estimates whether pairwise community dissimilarity differs from that expected by random chance alone. We used the re-scaling proposed by Chase et al. (2011) to obtain values that range from −1 to 1, and the significance was assessed using 10,000 simulations. A value of 0 denotes no difference in the observed dissimilarity from the null expectation (prevalence of stochastic factors); a value of 1 indicates a dissimilarity higher than expected by chance, while a value of −1 means that communities are less dissimilar than expected by chance. We considered a threshold value of >0.95 as statistical significance.

### Functional guilds

Functional categorization provides useful guides to navigate the complex fungal kingdom, confirming the positive effect of functional diversity on ecosystem processes (Crowther et al., 2014; Hättenschwiler, Tiunov & Scheu, 2005). To assign ecological functions of the identified ASVs, we used the FUNGuild v1.0 database (https://github.com/UMNFuN/FUNGuild; Nguyen et al., 2016), which presently represents the largest annotated databank for assigning fungal functional guilds (Xu et al., 2019).

Three confidence ranks suggesting the possibility of assumed guilds: “highly probable”, “probable”, and “possible” were evaluated through the comparison to the fungal database. “Highly probable” and “probable” guilds were selected to avoid the over-interpretation of ecological data. All taxa that did not match the database were classified as “unassigned” (Nie et al., 2018). In addition, guilds with a relative abundance <1% were clustered and assigned as “others.”

### Soil biogeochemical variables

Soil pH was measured in deionized water (1:2 w:v) with a digital pH meter (LAQUA-pH-1200-220 S). To allow nutrient concentrations and enzymatic activities to be corrected for soil sample moisture content, a 50 g subsample was oven-dried at 105 °C to constant weight for soil moisture determination using the gravimetric method (Schmugge, Jackson & McKim, 1980). Dissolved organic nutrients, nutrients in microbial biomass and available nutrients were extracted from moist samples (as Jones & Willett, 2006; Murphy & Riley, 1962; Robertson et al., 1999; Vance, Brookes & Jenkinson, 1987).

Inorganic nitrogen forms (NH4+ and NO3−) were extracted with 2 M KCl and determined colorimetrically by the phenol-hypochlorite method (Robertson et al., 1999). Inorganic phosphorus (iP) was extracted with sodium bicarbonate and was determined colorimetrically by the molybdate-ascorbic acid method (Murphy & Riley, 1962). Dissolved C, N and P were obtained as reported by Jones & Willett (2006). Total dissolved nitrogen and total dissolved phosphorus were digested by the macro-Kjendahl method (Bremmer, 1996). Next, dissolved organic carbon (DOC), nitrogen (DON) and phosphorus (DOP) were calculated as the difference between total dissolved forms and inorganic dissolved forms. Microbial C (Cmic), N (Nmic) and P (Pmic) concentrations were determined by the chloroform fumigation extraction method (as in Brookes et al., 1985; Lathja et al., 1999; Vance, Brookes & Jenkinson, 1987). Particularly, the Cmic was obtained as described by Joergensen (1996), and calculated by subtracting the extracted carbon in non-fumigated samples from that of fumigated samples and by dividing it by a KEC value (extractable part of microbial biomass C) of 0.45. The Nmic was obtained as described by Joergensen & Mueller (1996), and calculated as Cmic, but divided by a KEN value (extractable part of microbial biomass N after fumigation) of 0.54. The Pmic was obtained as described by Lathja et al. (1999) and including a fumigation-extraction technique with chloroform (Cole et al., 1978). Pmic was calculated as Cmic and Nmic and converted using a KP value (extractable part of microbial biomass P after fumigation) of 0.4 (Lathja et al., 1999). Finally, Cmic, Nmic and Pmic values were normalized on a dry soil basis.

All C forms were determined with a Total Carbon Analyzer (UIC Mod. CM 5012; Chicago, E.U.A), whereas the N and P forms were obtained by colorimetric analyses (Robertson et al., 1999; Murphy & Riley, 1962) using a Bran-Luebbe Auto Analyzer III (Norderstedt, Germany). Microbial P was analyzed by colorimetric analyses with a spectrophotometer (Genesys-20).

### Soil ecoenzymatic activity

We measured the activity of enzymes involved in the degradation of organic molecules with C, N and P in the nine soil subsamples, including three controls per enzyme (soil subsample without substrate). The analyzed enzymes included: β-1-4-glucosidase (GluSid), cellobiohydrolase (CeloBiHid), poliphenol oxidase (Lac), β-1-4-N-acetylglucosaminidase (GluMiniD), phosphomonoesterase (PMonEst), and phosphodiesterase (PDiEst). All enzymes were colorimetrically quantified using ρ-nitrophenol (ρNP) substrates with exception of Lac, where we used 2,2′-Azinobis (3-ethylbenzothiazoline-6-sulfonic acid)-diammonium salt (ABTS) as a substrate (Tabatabai & Bremner, 1969; Eivazi & Tabatabai, 1977; Eivazi & Tabatabai, 1988; Johannes & Majcherczyk, 2000; Verchot & Borelli, 2005). Experimental procedures were according to Tapia-Torres et al. (2015). Briefly, the tubes were centrifuged after the incubation period and then 750 μl of supernatant was diluted in a final volume of three ml of deionized water and the absorbance of ρ-nitrophenol (ρNP) at 410 nm and 460 nm for ABTS was measured in a Genesys 20 spectrophotometer. The enzyme activity was calculated by subtracting the absorbance values of the controls (sample and substrate) from the absorbance values of the soil samples to finally obtain an average for the triplicates per soil sample. The activity was expressed as micromoles of ρNP released per gram of dry weight soil per hour (μmol ρNP [g SDW] -1 h-1) for substrate containing ρNP or Tyrosine released per gram of dry soil per hour (nmol Tyr [g SDW]) for Lac.

### Multivariate analyses

We evaluated species responses to environmental variables by means of a redundancy analysis (RDA) using raw species abundances and the environmental data. To select the best set of variables that adjust to the model and to avoid multicollinearity, we conducted a variance inflation factor analysis, retaining those variables with values <10. Next, we used the ordiR2step function with 10,000 permutations, conserving variables with *p* values < 0.01. To test the significance of the adjusted model, we performed an ANOVA, estimating the r2 using the RsquareAdj function. To evaluate whether environmental differences explain taxonomic composition per subsample, we performed Mantel tests exploring the correlation between taxonomic composition (Sorensen and Bray–Curtis dissimilarity matrices) and environmental Euclidean matrices (soil ecoenzymatic activity and biogeochemical variables); the significance was assessed through 10,000 replications (Borcard, Gillet & Legendre, 2018). All of the above-mentioned multivariate analyses were conducted using the R package “vegan” v2.5-6 (Oksanen et al., 2019).

## Results

### Evaluation of soil fungal taxonomic diversity

A total of 682,361 raw ITS1 reads were obtained from the Illumina MiSeq sequencing platform. After the filtering and denoising processes (removing of 178, 089 reads), we obtained a total of 504, 272 ITS1 assembled sequences. We inferred a total of 1992 (355,821 sequences) fungal ASVs, ranging from 89,502 sequences (in subsample A1) to 10,835 sequences (in subsample C2) across samples, and collapsing into 396 ASVs at the species level. All of the accumulation curves for individual (nine) subsamples gradually approached saturation plateau (Fig. S1), indicating that our sequencing depth was sufficient.

**Figure 1:**
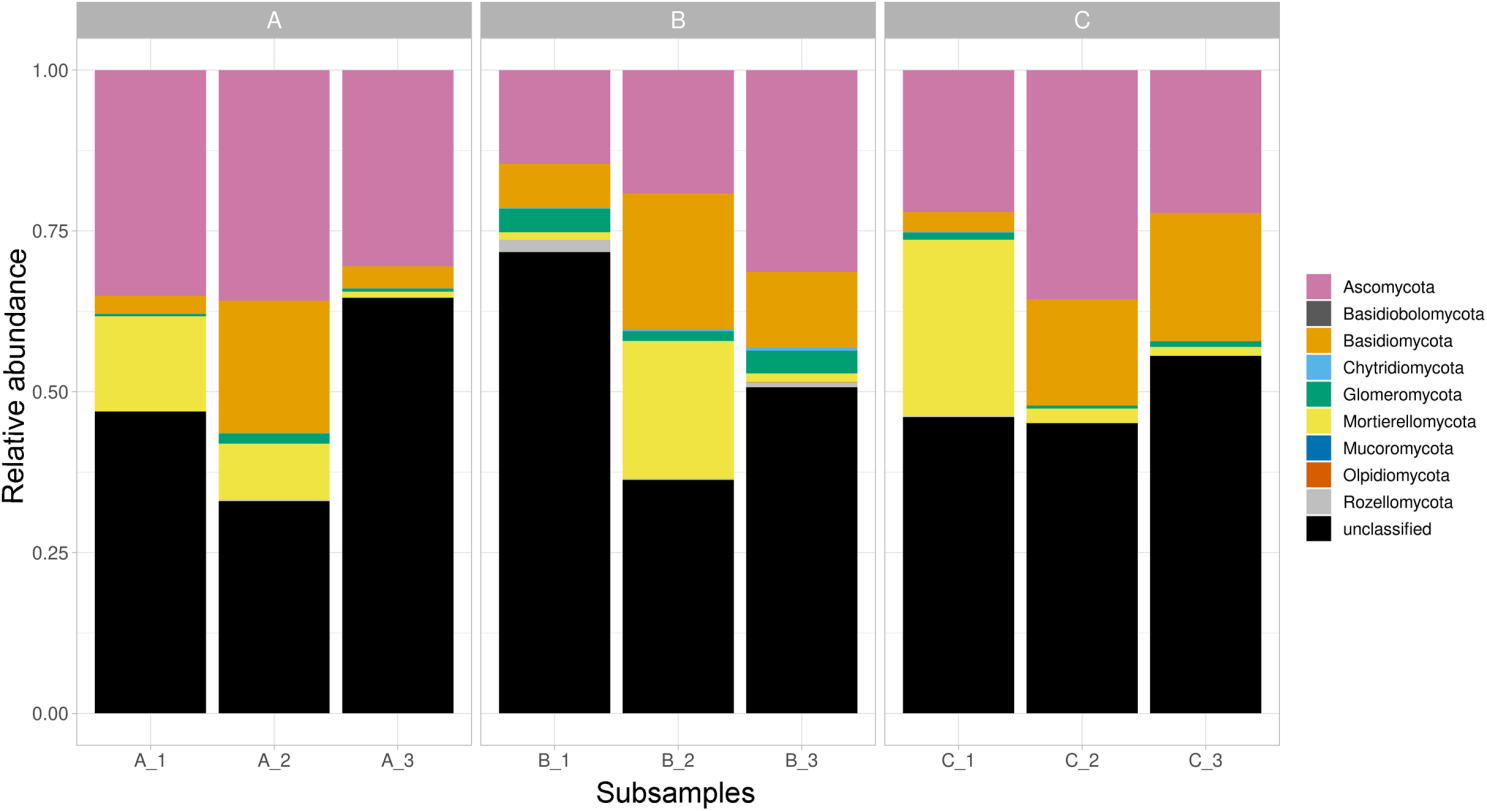
Relative abundance of fungal phyla across subsamples. Three sampling sites are indicated at the top.

Fungal ASVs belonged to nine phyla (*Ascomycota*, *Basidiobolomycota*, *Basidiomycota*, *Chytridiomycota*, *Glomeromycota*, *Mortierellomycota*, *Mucoromycota*, *Olpidiomycota*, and *Rozellomycota*), 22 classes, 58 orders, 119 families, and 171 genera. Within these phyla, Ascomycota was the most abundant and diverse phylum, accounting for 735 ASVs; followed by, *Basidiomycota* (273 ASVs), *Glomeromycota* (61 ASVs), *Mortierellomycota* (42 ASVs), *Chytridiomycota* (11 ASVs), *Mucoromycota* (3 ASVs), *Olpidiomycota* (1 ASV), and *Basidiobolomycota* (1 ASV); in addition to *Rozellomycota* (17 ASVs; Table S1). Furthermore, 42.5% of the reads corresponded to unclassified phyla. *Mortierellomycota*, *Glomeromycota*, and *Ascomycota* were detected in all the sites varying in their relative abundance (Fig. 1).

The ASVs that belonged to the *Ascomycota* were classified into 9 classes, with the *Sordariomycetes* as the most diverse component (242 ASVs), followed by *Leotiomycetes* (181 ASVs), *Dothideomycetes* (59 ASVs), and *Eurotiomycetes* (50 ASVs). Within *Basidiomycota*, *Agaricomycetes* (212 ASVs) was the richest class, followed by *Tremellomycetes* (20 ASVs). Sequences of the *Chytridiomycota* were affiliated with the *Chytridiomycetes* and *Rhizophydiomycetes* (2 and 3 ASVs respectively). Sequences of the *Glomeromycota* were principally affiliated to the *Glomeromycetes* (34 ASVs); whereas *Basidiobolomycota* was represented by the class *Basidiobolomycetes* (1 ASV). Sequences within *Mortierellomycota* were comprised within the *Mortierellomycetes* (42 ASVs), and *Olpidiomycota* within the lineage GS18 (1 ASV). Sequences of the *Mucoromycota* were referred to the *Endogonomycetes* (3 ASVs; Fig. S2A). We identified 58 classes, with the *Mortierellales*, *Helotiales* and *Hypocreales* as dominant components (Fig. S2B).

Nearly 36.5% of the sequences were classified at the family level (119 ASVs). The most abundant family was *Mortierellaceae* (36,817 sequences), followed by *Hypocreaceae* (10,085 sequences), *Nectriaceae* (8,261 sequences), and *Russulaceae* (6,465 sequences), *Archaerhizomycetaceae* (5,456 sequences). At the genus level, 171 fungal ASVs were identified (17.5% of the sequences), among which top abundant taxa were: *Mortierella* (28,895 sequences), *Trichoderma* (10,059 sequences), *Ilyonectria* (7,572 sequences), *Russula* (6,450 sequences), *Archaeorhizomyces* (5,456 sequences), *Sistotremastrum* (2,311 sequences), *Clavulina* (1,406 sequences), *Apiotrichum* (1,029 sequences), *Tolypocladium* (977 sequences), *Hymenosciphus* (641 sequences), *Leuhumicola* (639 sequences), *Cephalotheca* (610 sequences), *Chaetosphaeria* (519 sequences), *Chloridium* (509 sequences), *Ganoderma* (430 sequences), *Nadsonia* (400 sequences), *Pezoloma* (387 sequences), *Dissophora* (372 sequences), *Terramyces* (299 sequences), *Dendrosporium* (295 sequences), *Submersisphaeria* (259 sequences), *Penicillium* (246 sequences), *Leptodontidium* (237 sequences), *Pseudeurotium* (219 sequences), and *Neonectria* (201 sequences) (Table S1).

**Figure 3:**
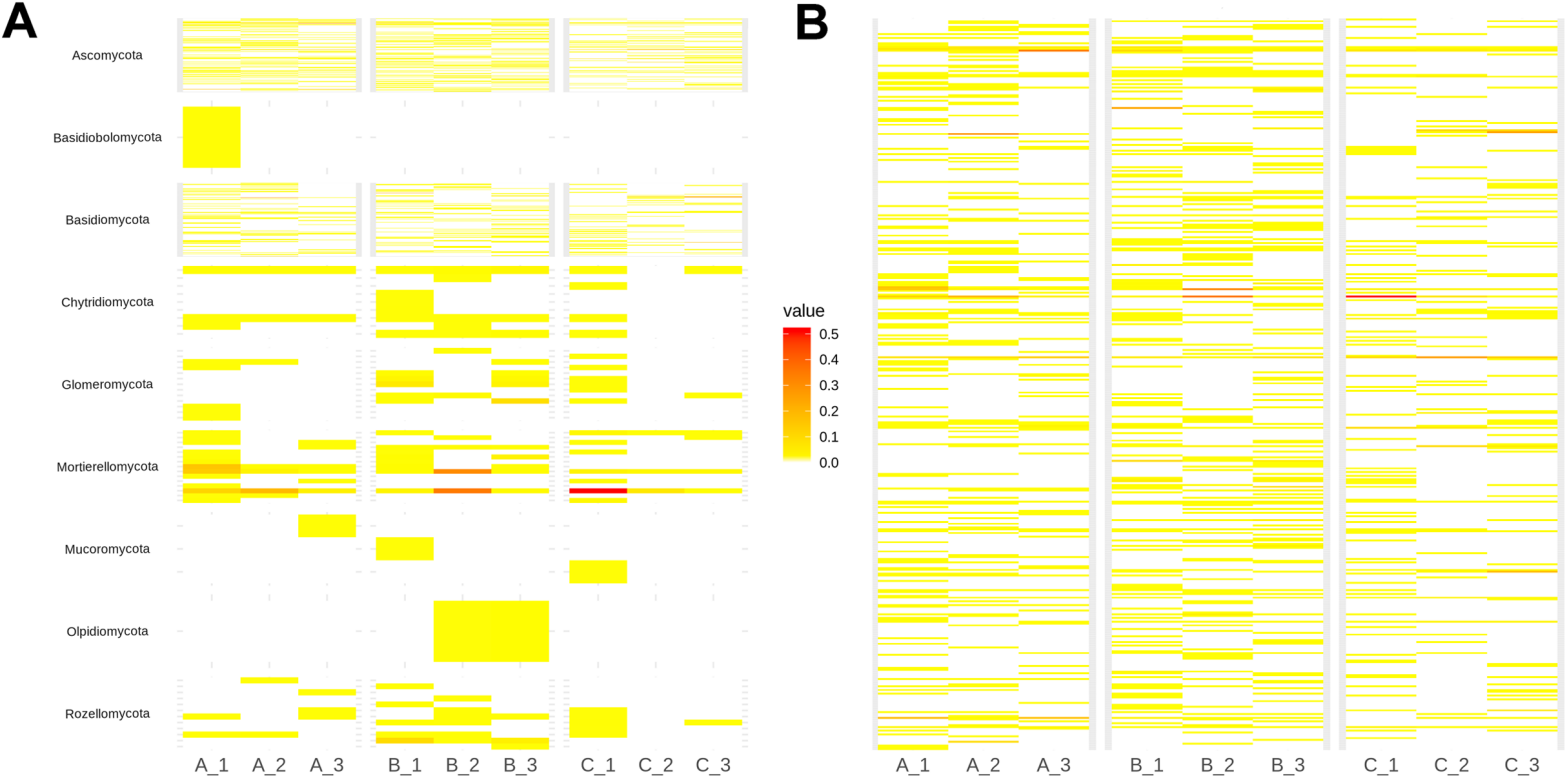
Heatmap showing soil fungal richness and abundance at the (A) phyla, and (B) species level across subsamples. The x-axis indicates subsamples and the y-axis fungal taxa.

At a species level, around 81% of the taxa were rare (<100 reads; Fig. 3B). Top abundant species (>650 reads, upper 95% quantile) were: *Mortierella* sp.1 (17,448 sequences), *Ilyonectria* sp. (7,572 sequences), *Mortierella* sp. 2 (7,123 sequences), *Trichoderma* sp. 1 (5,366 sequences), *Archaeorhizomyces* sp. 5 (5,091 sequences), *Mortierella* sp. 3 (3,358 sequences), *Russula* sp. 1 (3,254 sequences), *Trichoderma* sp. 2 (2,622 sequences), *Sistotremastrum* sp. (2,311 sequences), *Trichoderma* sp. 3 (2,032 sequences), *Russula* sp. 2 (1,929 sequences), *Helotiales* sp. 2 (1,911 sequences), *Clavulina* sp. (1,406 sequences), *Russula* sp. 3 (1,144 sequences), GS11 (1,131 sequences), *Agaricales* sp. 1 (1,100 sequences), *Apiotrichum* sp. (1,029 sequences), *Helotiales* sp. 1 (884 sequences), *Tolypocladium* sp. 1 (683 sequences) and *Leucosporidiales* sp. (653 sequences; Table S1).

### Alpha and beta diversity patterns

Richness ranged from 86 (in subsample C2) to 127 ASVs at the species level (in subsample B1). Shannon diversity index showed a remarkably high alpha diversity at a small-scale, ranging from 4.04 in B3 to 2.08 in C1. In addition, evenness values demonstrated a strong dominance in the community, fluctuating between 0.47 in B3 and 0.09 in C1 (Table 1).

**Table 1:** Soil fungal alfa diversity estimates across the nine subsamples.

Sites	Subsamples	Counts	Richness	Shannon index	Evenness
A	A1	125	125	2.8	0.1
	A2	114	114	2.7	0.1
	A3	78	78	2.5	0.2
B	B1	127	127	3.2	0.2
	B2	117	117	2.4	0.1
	B3	120	120	4.0	0.5
C	C1	86	86	2.0	0.1
	C2	58	58	3.0	0.3
	C3	70	70	2.6	0.2

**Figure 2:**
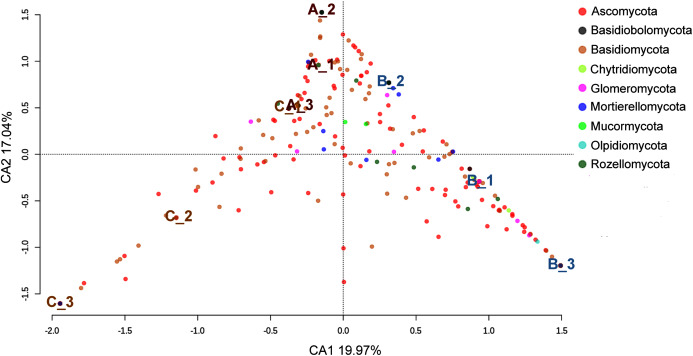
Spatial ordination of the fungal community at the phylum level across the nine subsamples (excluding the unclassified component). Sampling sites are indicated as follows: A = red labeling, B = blue labeling, C = orange labeling.

As shown in the CCA, subsamples aggregated in agreement with their origin (first and second components accounting for 19.97% and 17.04% of the total variation respectively). For instance, subsamples A1, A2, and A3 grouped together, differentiating from subsamples B1, B2 and B3. We observed the overlap of subsamples A3 and C1 (Fig. 2). Despite major fungal phyla were uniformly distributed, some phyla such as *Basidiobolomycota* was restricted to subsample A1, and *Olpidiomycota* to subsamples B2 and B3 (Fig. 3A). Similarly, the Sorensen PERMANOVA results (*p* ≤ 0.001) indicated community clustering based on presence-absence data; in contrast to Bray-Curtis PERMANOVA results (*p* = 0.24), where no significant relationship between fungal abundances and the ordination of sites was detected.

**Figure 4:**
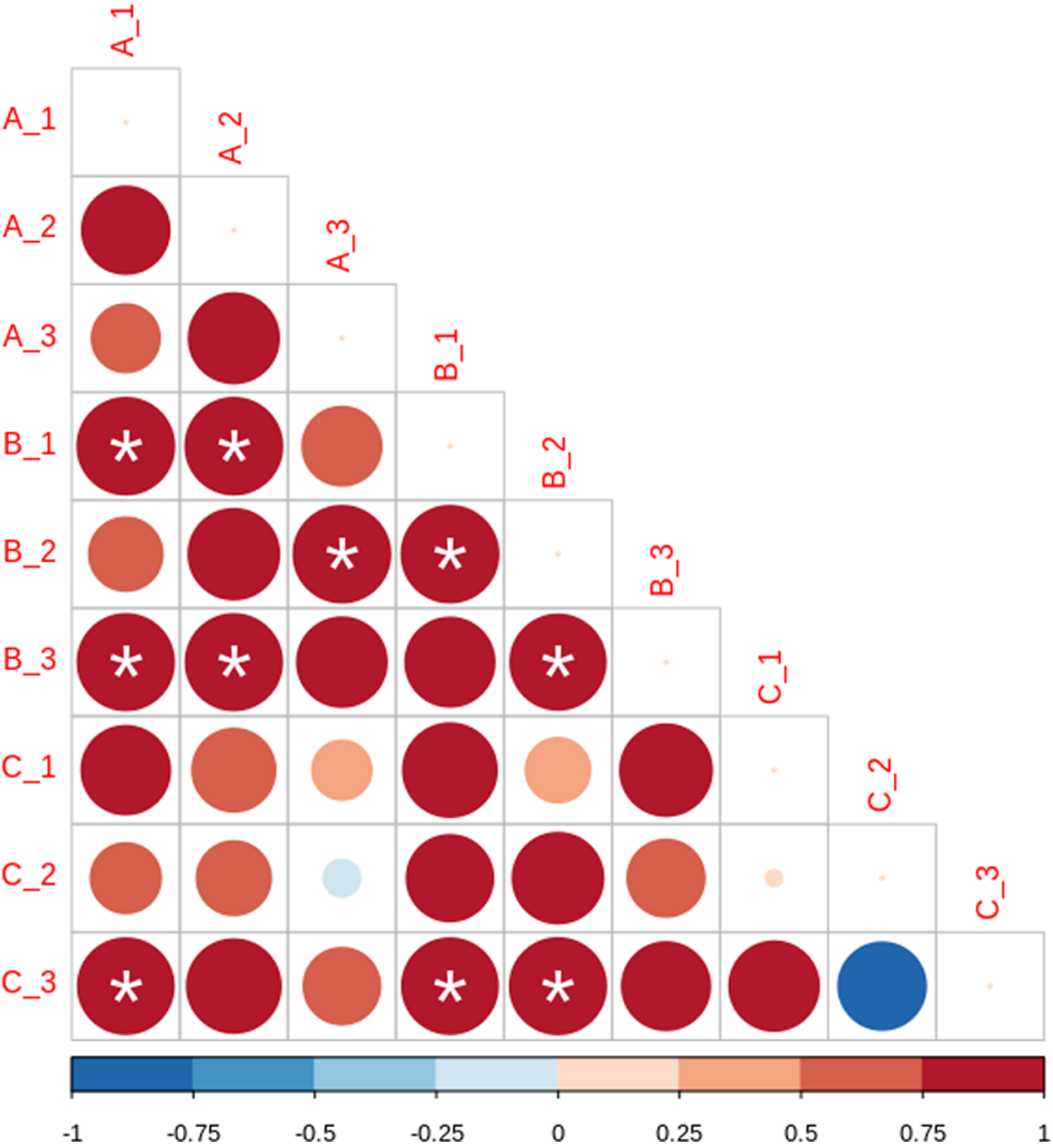
Lower matrix of the paired probabilistic Raup–Crick measures across subsamples. Blue indicates that communities are less dissimilar than expected by chance, and red dissimilarity higher than expected by chance. Asterisks denote significant values (*p* < 0.05). The size and color intensity of the circles is proportional to the Raup-Crick metrics as indicated in the horizontal lower bar.

Based on beta diversity estimates we detected a high small-scale variation among the nine subsamples, with Bray–Curtis distances varying between 0.5887 for A1 and B2, and 0.9208 for B3 and C1 (Table 2). In addition, Sorensen values ranged from 0.7297 for A2 and B2, and 0.8584 for B3 and C3 (Table 3). Lastly, Raup–Crick metric results showed significant positive dissimilarity values, predominantly in site B (Fig. 4).

**Table 2:** Bray–Curtis beta diversity estimates across the nine subsamples.

	A1	A2	A3	B1	B2	B3	C1	C2	C3
A1	0.00								
A2	0.62	0.00							
A3	0.70	0.75	0.00						
B1	0.80	0.79	0.62	0.00					
B2	0.59	0.63	0.88	0.87	0.00				
B3	0.92	0.89	0.84	0.78	0.85	0.00			
C1	0.67	0.70	0.74	0.82	0.66	0.92	0.00		
C2	0.86	0.81	0.65	0.81	0.85	0.75	0.85	0.00	
C3	0.85	0.82	0.76	0.86	0.90	0.88	0.85	0.62	0.00

**Table 3:** Sorensen beta diversity estimates across the nine subsamples.

	A1	A2	A3	B1	B2	B3	C1	C2	C3
A1	0.00								
A2	0.73	0.00							
A3	0.76	0.78	0.00						
B1	0.73	0.79	0.82	0.00					
B2	0.79	0.73	0.82	0.76	0.00				
B3	0.82	0.82	0.83	0.73	0.75	0.00			
C1	0.76	0.81	0.81	0.78	0.79	0.83	0.00		
C2	0.83	0.82	0.83	0.82	0.85	0.85	0.83	0.00	
C3	0.84	0.83	0.81	0.81	0.85	0.86	0.81	0.75	0.00

### Fungal community and small-scale spatial structure

**Figure 5:**
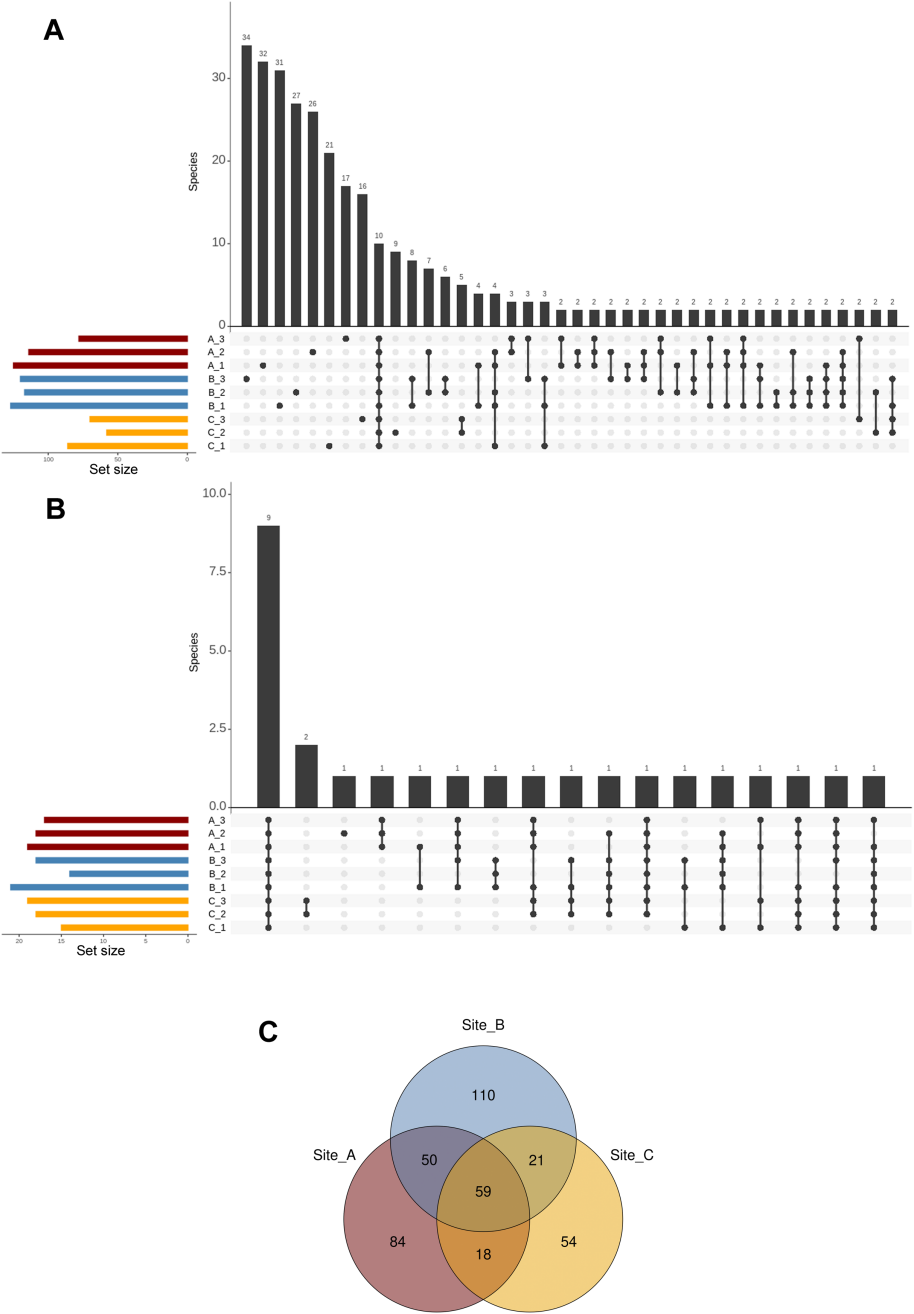
Shared ASVs across the nine subsamples. Number of ASVs at the species level (A), and top abundant ASVs at the species level (B) that are shared across the nine subsamples (stacked in the x-axis); and Venn diagram showing the number of shared ASVs at the species level across the three sampling sites (C).

We defined the core fungal mycobiome based on the co-occurrence of species across all soil subsamples. Our results indicated that only ten taxa (from a total of 396 ASVs at the species level) were shared among the nine subsamples, while most of the species (213 taxa) occurred in a single subsample (Fig. 5A). In addition, the analysis of the top abundant ASVs (>650 number of sequences, upper 95% quantile) revealed that only nine species were shared among the nine subsamples (Fig. 5B). Complementarily, at the site level our results showed that 59 species were shared across the three sites, whereas 84, 110, and 54 species were restricted to sites A, B, and C respectively (Fig. 5C).

### Functional guilds

**Figure 6:**
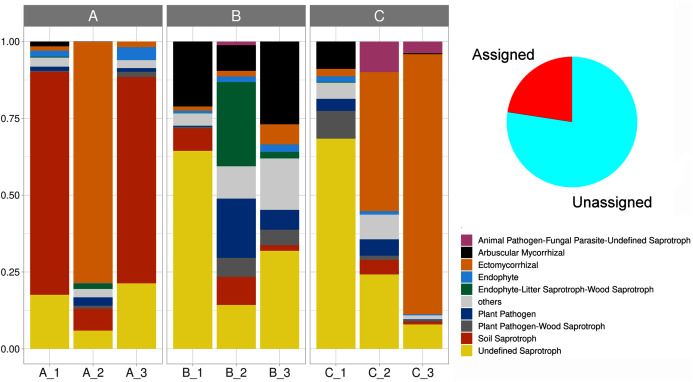
Stacked barplot showing the relative proportion (y-axis) of the assigned soil fungal functional guilds (shown in red in the pie chart) across subsamples (x-axis). The three sampling sites are indicated horizontally at the top.

We delimited 10 soil fungal functional guilds, with an uneven distribution among subsamples. Overall, undefined saprotrophs were the most diverse element, with 74 species (6,790 sequences); followed by ectomycorrhizal with 35 species (8,150 sequences), wood saprotrophs with 15 species (199 sequences). Plant pathogens accounted for 14 species (707 sequences), arbuscular mycorrhizal with 13 species (1,737 sequences), plant pathogen-wood saprotrophs with eight species (447 sequences), and soil saprotrophs with five species (5,456 sequences). Thirteen life strategies (bryophyte parasite-litter saprotroph-wood saprotroph, dung saprotroph, dung saprotroph-plant saprotroph-wood saprotroph, ectomycorrhizal-wood saprotroph, endophyte-plant pathogen-undefined saprotroph, endophyte-plant pathogen-wood saprotroph, fungal parasite-lichen parasite, fungal parasite-undefined saprotroph, leaf saprotroph, leaf saprotroph-plant pathogen-undefined saprotroph-wood saprotroph, orchid mycorrhizal, plant pathogen-undefined saprotroph, undefined saprotroph-wood saprotroph) represented the oddest guild (frequency < 1%), being clustered and hereafter defined as “others”. Lastly, 70.8% of the reads were affiliated with an unassigned functional group (Fig. 6, Table S2).

### Soil biogeochemical and ecoenzymatic variables

**Figure 7:**
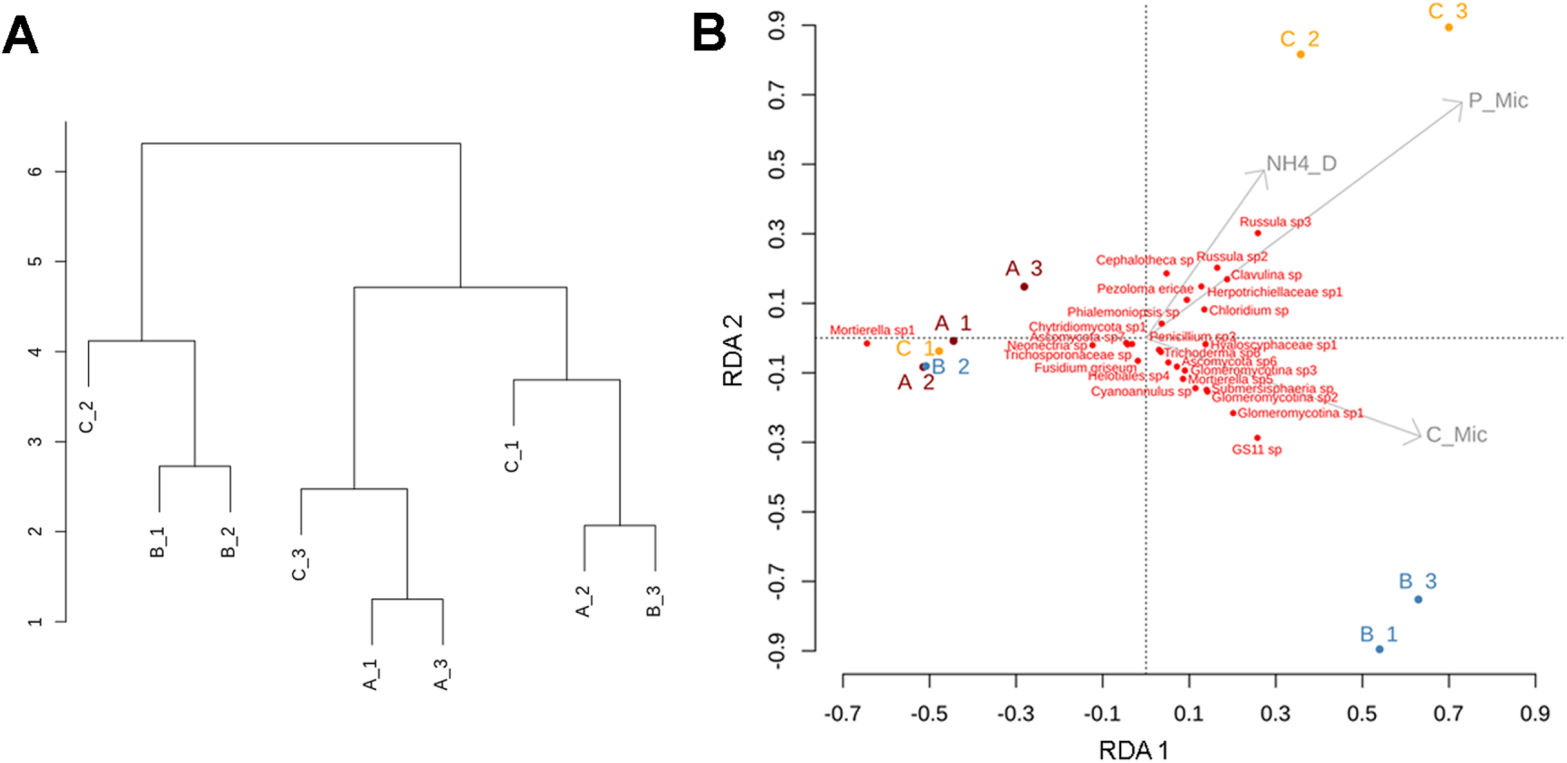
Clustering patterns of the nine subsamples. Clustering patterns of the nine subsamples based on Euclidean distances of the biogeochemical variables (A), and redundancy analysis (RDA) plot depicting the relationship between the nine subsamples, soil nutrient concentrations (microg/g-1), and soil fungal diversity (B), where NH4_D = disolved ammonium, P_Mic = microbial phosphorous, C_Mic = microbial carbon. Sampling sites are indicated as follows: A = red labeling, B = blue labeling, C = orange labeling.

Our soil biogeochemical results (based on the analysis of the retained variables: pH, COD, NOD, NH4_D, Cmic, Nmic, and Pmic) showed a high environmental heterogeneity between sites and subsamples, with sparse clustering patterns, especially for site C (Fig. 7A). The pH values ranged between 4.4 in A1 and 5.2 in C1. The total nutrient concentration ranged as follow: the lower value of total carbon was 16.6 mg/g^−1^ and was observed in C1 meanwhile, the higher concentration was 75.6 mg/g^−1^ in B2; the total nitrogen concentration range was 1.68–4.2 mg/g^−1^ in C1 and B2 respectively; and the lower total phosphorous concentration was 0.09 mg/g^−1^ in C1 and 0.25 mg/g^−1^ in B2. Data associated to DOC showed the lower value in B3 (52.5 μg/g^−1^) and higher value in B1 (287 μg/g^−1^), the DON concentration ranged between 2.6 μg/g^−1^ in C2 and 9.8 μg/g^−1^ in A2; the DOP concentration ranged between 0.87 μg/g^−1^ in A3 and 5.46 μg/g^−1^ in C2. The ammonium concentration ranged between 3.2 μg/g^−1^ in A2 and 27.5 μg/g^−1^ in C2. The nutrient concentration associated to microbial biomass followed the same heterogeneity than soil nutrient concentrations. The Cmic ranged between 148 μg/g^−1^ in C1 and 1,215 μg/g^−1^ in B1; Nmic ranged between 12 μg/g^−1^ in C3 and 96.61 μg/g^−1^ in C2; and the lower Pmic concentration observed was 9.2 μg/g^−1^ in A2 and the higher concentration was 48.2 μg/g^−1^ in C2 (Table S3).

The subsamples from site C showed the lowest values of GluSid and GluMiniD, clearly differentiating from A and B. The highest values of CeloBiHid were obtained in all of the subsamples from site B. The activities of Lac and PMonEst were heterogeneous across subsamples, with the highest values occurring in B1 followed by B2. With respect to PDiEst, the highest value was observed in B2, followed by C3, and the lowest value was detected in A1 followed by C1 (Table S3).

### Multivariate analysis

The RDA (involving environmental variables and species data) confirmed the sparse clustering of subsamples in relation to sampling sites. This pattern was evident in the subsamples from site C, since C1 grouped with A1, A2, A3, and B2 being mostly explained by the presence of *Mortierella* sp. Whereas, C2 and C3 were related with microbial phosphorus, dissolved ammonium, and fungal taxa such as *Cephalotheca* sp., *Chloridium* sp., *Clavulina* sp. (potentially ectomycorrhizal fungi), *Herpotrichiellaceae* sp., *Pezoloma ericae* (ericoid mycorrhizal fungus), and *Russula* spp. 2, 3 (ectomycorrhizal fungi). In addition, subsamples B1 and B3 were associated with microbial C, and taxa such as *Glomeromycotina* spp. 1, 2, and 3 (theoretically responsible for arbuscular mycorrhizas), GS11 (divergent lineage of *Rozellomycota* that is relatively more common in cool temperate climate; Tedersoo et al., 2018), and *Submersisphaeria* sp., and *Trichoderma* sp. 6 (Fig. 7B).

The Mantel tests revealed a statistically significant correlation between taxonomic composition and enzymatic activity (Sorensen *p* = 0.016, r = 0.461; Bray–Curtis *p* = 0.063, r = 0.270), yet no correspondence with the biogeochemical variables (Sorensen *p* > 0.330, r = 0.072; Bray–Curtis *p* = 0.444, r = 0.021).

## Discussion

### Fungal megadiversity at the small-scale

The total number of 396 identified soil fungal ASVs at a species level within a 10 × 10 × 10 m triangular transect, far exceeds previous direct observation-based figures at the local scale for MCF in the Veracruz State (most extensively explored region in the country) with 355 species (Arias, Heredia-Abarca & Castañeda-Ruiz, 2018); and represents near a third of the 1,274 fungal species reported at the large-scale for Mexico (del Olmo-Ruiz et al., 2017). It is worth noting that this may be attributed to methodological biases in previous reports, which are restricted to the direct observation and/or isolation of fungi, coupled with a morphology-based taxonomic identification. This approach presents a number of limitations, such as: the incapability of many microorganisms (actively developing in nature) to grow in artificial culture media in *ex situ* conditions, overgrowth of dominant taxa inhibiting the development of slow growing species, fungal selection/observation, the ability of species to sporulate in culture, among others (Ozerskaya et al., 2009; Korpelainen, Pietiläinen & Huotari, 2016). Furthermore, the herein reported richness represents 5.19% of the total number of OTUs (clustered at the 97%) in soil samples from 17 forests (boreal, temperate, subtropical and tropical forests), along a latitudinal transect (Shi et al., 2014); and 2.65% from a large-scale investigation in 365 forest plots covering five climate zones (Hu et al., 2019). Overall, this evidences the magnitude of soil fungal species richness in MCFs, and the need for culture-independent approaches to transcend the limitations of direct observation and cultivation methods.

Likewise, higher-rank taxonomic diversity surpassed previously known numbers at the large-scale for Mexico with nine phyla (*Ascomycota*, *Basidiobolomycota*, *Basidiomycota*, *Chytridiomycota*, *Glomeromycota*, *Mortierellomycota*, *Mucoromycota*, *Olpidiomycota*, and *Rozellomycota*) in contrast to five (*Ascomycota*, *Basidiomycota*, *Chytridiomycota*, *Glomeromycota*, *Zygomycota*); and 22 classes *versus* 18 (del Olmo-Ruiz et al., 2017). In addition, this represents the first record of the basal fungal clades (circumscribed *a posteriori* in Tedersoo et al., 2018) such as *Basidiobolomycota*, *Mortierellomycota*, *Mucoromycota*, *Olpidiomycota*, and *Rozellomycota* for Mexican MCF. This suggests an extraordinarily high taxonomic diversity at a small spatial scale, and portrays the need of integrated studies and conservation strategies considering high fungal diversity patterns at this scale.

Top abundant taxa were mostly represented by soil-borne emblematic microfungal species. Particularly, saprotrophic and root endophytes such as *Mortierella* spp. (Summerbell, 2005) dominated in all the subsamples. Further copious taxa included *Ilyonectria* spp., known to occur as endophytes in healthy plants (inhibiting fungal root pathogens; White, Chilvers & Evans, 1962), and root pathogens on a number of herbaceous and woody host plants (Cabral et al., 2012). Likewise, mycoparasites such as *Trichoderma* (Guzmán-Guzmán et al., 2019), root-associated elements such as *Archaeorhizomyces* (Leech et al., 2020), and entomopathogenic species such as *Tolypocladium* (Samson & Soares, 1984) were highly abundant, suggesting that synergistic and antagonist biotic interactions may act as major drivers of soil small-scale community structuring processes.

The Shannon index evidenced a high alpha diversity in all the subsamples, in agreement with former reports accounting MCFs as macroorganismal biodiversity hotspots (Bubb et al., 2004). Our result postulates MCFs as soil microbial hotspots that host diverse fungal communities denoted by a few highly dominant and widespread taxa (low community evenness), and a large majority of rare species (hereafter referred as the rare biosphere, *sensu* Sogin et al., 2006) lacking clear spatial-environmental clustering patterns at small spatial scale. Presumably, this rare biosphere may act as a “seed bank” that thrives during a different season or under fluctuating conditions (favoring the proliferation of individual taxa); whereas the dominant component, seemingly more active, may be defined as the core mycobiome (Fuhrman, 2009).

### Microspatial ecological patterns of soil fungal diversity

The observed high beta diversity estimates, sparse clustering of subsamples based on environmental distances, patchy ecoenzymatic activities, and the RDA, revealed remarkably high microhabitat heterogeneity within neighboring subsamples (1 m-distance). This pattern was expressly evident for site C, which showed the highest environmental variability. This may be due either to *in situ* non-uniform addition of organic material or variations in temperature and moisture (Christ & David, 1996).

Even though spatial heterogeneity prevailed in our study system, we also detected site-specific fungal assemblages and functional signatures, where deterministic processes dominated as shown by the Raup-Crick test. For instance, the samples from site A showed the lowest values of GluSid, GluMiniD and Cmic, being dominated by soil saprotrophs (mainly *Ascomycota*, *Basidiomycota*, and *Mortierellomycota*). In contrast, the samples of site B showed higher nutrients concentration and a profuse occurrence of functional guilds such as arbuscular mycorrhiza, undefined saprotrophs, endophyte-litter saprotrophs, and phytopathogens (*Ascomycota*, *Basidiomycota*, *Mortierellomycota*, *Glomeromycota*, and *Rozellomycota*). Under this scenario, environmental filtering may operate as a major force modeling soil fungal assemblages at a small-scale, and implying micro-environmental conditions and fungal metabolic specialization (Nuccio et al., 2020). In addition, the RDA supported the relationship between soil nutrients and the taxonomic composition of the fungal community at the small-scale in accordance to further reports (*e.g*. isolated tree islands, and Glassman, Wang & Bruns, 2017; alpine tundra, Ni et al., 2018). So, at large these results are crucial for MCF conservation and management, since they diverge from broad spatial-scale fungal patterns, where neutral processes (*e.g*. dispersal limitation) are known to prevail (Cline & Zak, 2014; Feinstein & Blackwood, 2013; Martiny et al., 2006; Talbot et al., 2014; Wu et al., 2013).

### Functional guilds

Soil as a fundamental and irreplaceable natural resource, governs plant productivity and sustains terrestrial biogeochemical cycles (Nannipieri et al., 2003). These capacities are linked to its belowground functional microbial diversity (Jansson & Hofmockel, 2018), which may show spatial patterns in response to differences in litter quality and microclimate in areas under trees (Mungai et al., 2005). We approximated a high fungal functional diversity (10 fungal guilds) at the small-scale, which reinforces our findings of a high alfa- and beta-diversity estimates in the same sites. Moreover, the observation of saprobes as the larger functional guild in soil, agrees with previous work at the large scale suggesting these fungi are key players in MCF nutrients cycling (Looby & Treseder, 2018). In addition, the high abundance of fungal lineages implicated in nitrogen dynamics such as *Glomeromycota* and *Mucoromycota*, advocate N-mediated rhizopheric fungal interactions with plants (Bonfante, 2020).

We detected small-scale spatial patterns, differentiating functional guilds neighboring the rhizosphere of iconic MFC plant species. For instance, the soil near *O. mexicana* was plainly dominated by soil saprobes followed by ectomycorrhiza; in contrast to the soil adjacent to the arborescent fern *A. salvinii* where functional guilds were evenly represented including undefined saprotrophs, arbuscular mycorrhiza, endophytes and plant pathogens. However, our field design (including a single representative of each plant species) hampers interpretations on host-mycobiome associations. So, these results should be further properly examined in forthcoming studies addressing this constraint in sample size.

Soil ecoenzymatic results exposed distinct functional processes linked to nutrients turn over at the small-scale. Overall, these results confirmed that soil fungal diversity might be concurrently responding to micro-environmental conditions. In agreement with the observed microspatial ecological patterns, evidencing a multifactorial environmental filtering in the assemblage of fungal communities (Lucero et al., 2006), epitomized by a highly heterogeneous abiotic setting. This should represent a central element in terms of the MCF conservation agenda, considering abiotic variations at the small-scale upon which evolution will play out and support many actors (Lawler et al., 2015).

MCFs have slow organic matter decomposition and nutrient cycling rates (Bruijnzeel et al., 1993; Grubb, 1977), as a result of abundant rainfalls, cool temperatures, reduced overall photosynthesis (given the minor light incidence), and low evapotranspiration (due to high humidity; Schawe et al., 2010). Consequently, this system stores more soil C than lowland forests (Grieve, Proctor & Cousins, 1990; Raich et al., 2006; thereby contributing to climate change mitigation), being particularly susceptible to climate change (Bradford et al., 2016). We speculate that the high abundance of saprotrophs supports former postulates on MCF soil functional modifications under climate change, where (1) global C cycling changes occur as a result of exponential increases in decomposition rates with higher temperatures, releasing CO_2_ from the soil (Benner, Vitousek & Ostertag, 2010); and (2) selected fungal groups such as pathogens and wood saprotrophs proliferate (Looby & Treseder, 2018; Pounds, Fogden & Campbell, 1999; Pounds et al., 2006). In this setting, additional studies are required for a better understanding of the mechanisms underpinning directional species turnover in other systems with similar environmental conditions.

## Conclusions

Our data evidenced a taxonomically and functionally diverse fungal community within a 10 m-triangular transect, outnumbering previous figures for Mexico and representing the 13.4% of the overall fungal diversity reported for Neotropical MCFs. This supports our hypothesis that MCF harbors a highly diverse fungal community at the small-scale. The uneven co-occurrence of numerous functional guilds across a 10 m-triangular transect may indicate resource-based small-scale niche partitioning processes (where different resource requirements across taxa facilitates specialization on particular patch types), and could be further investigated to address soil-related axes of specialization. Moreover, our results suggested that mainly deterministic assembly processes operate at a small spatial scale, under an extraordinarily heterogeneous environmental setting. Nevertheless, patchy ecoenzymatic activities and guilds aggregation patterns denoted a dominance of characteristic functional fungal entities at the site level, suggesting selected fungal metabolic capacities to access local nutrients.

## Supplemental Information

10.7717/peerj.11956/supp-1 Supplemental Information 1 Reads of the identified fungal ASVs from soil samples in a pristine montane cloud forest.

10.7717/peerj.11956/supp-2 Supplemental Information 2 Identified fungal functional guilds from soil samples collected in a pristine montane cloud forest.

10.7717/peerj.11956/supp-3 Supplemental Information 3 Biogeochemical variables, stoichiometric ratios and soil enzymatic activities.

10.7717/peerj.11956/supp-4 Supplemental Information 4 Rarefaction curves. Accumulation curves of community richness estimates at different sampling sites (A, B, C) and subsamples (1, 2, 3).

10.7717/peerj.11956/supp-5 Supplemental Information 5 Barplot showing soil fungal composition at order level class level (A), and order level (B), y-axis indicates ASV counts.

## Additional Information and Declarations

### Field Study Permissions

The following information was supplied relating to field study approvals (*i.e*., approving body and any reference numbers): Samplings were conducted under the permit by the Secretaría de medio Ambiente y Recursos Naturales, Subsecretaría de Gestión para la Protección Ambiental, Dirección General de Gestión Forestal y de Suelos, issued by the Mexican government (SGPA/DGGFS/712/2955/17).

### Data Availability

The following information was supplied regarding data availability: Data are available at NCBI GenBank: PRJNA634568.
