# Supplementary figures and images for: Small-scale variation in a pristine montane cloud forest: evidence on high soil fungal diversity and biogeochemical heterogeneity
Source: PeerJ. 2021 Aug 11;9:e11956. doi: 10.7717/peerj.11956 (PMC8364316; doi:10.7717/peerj.11956)

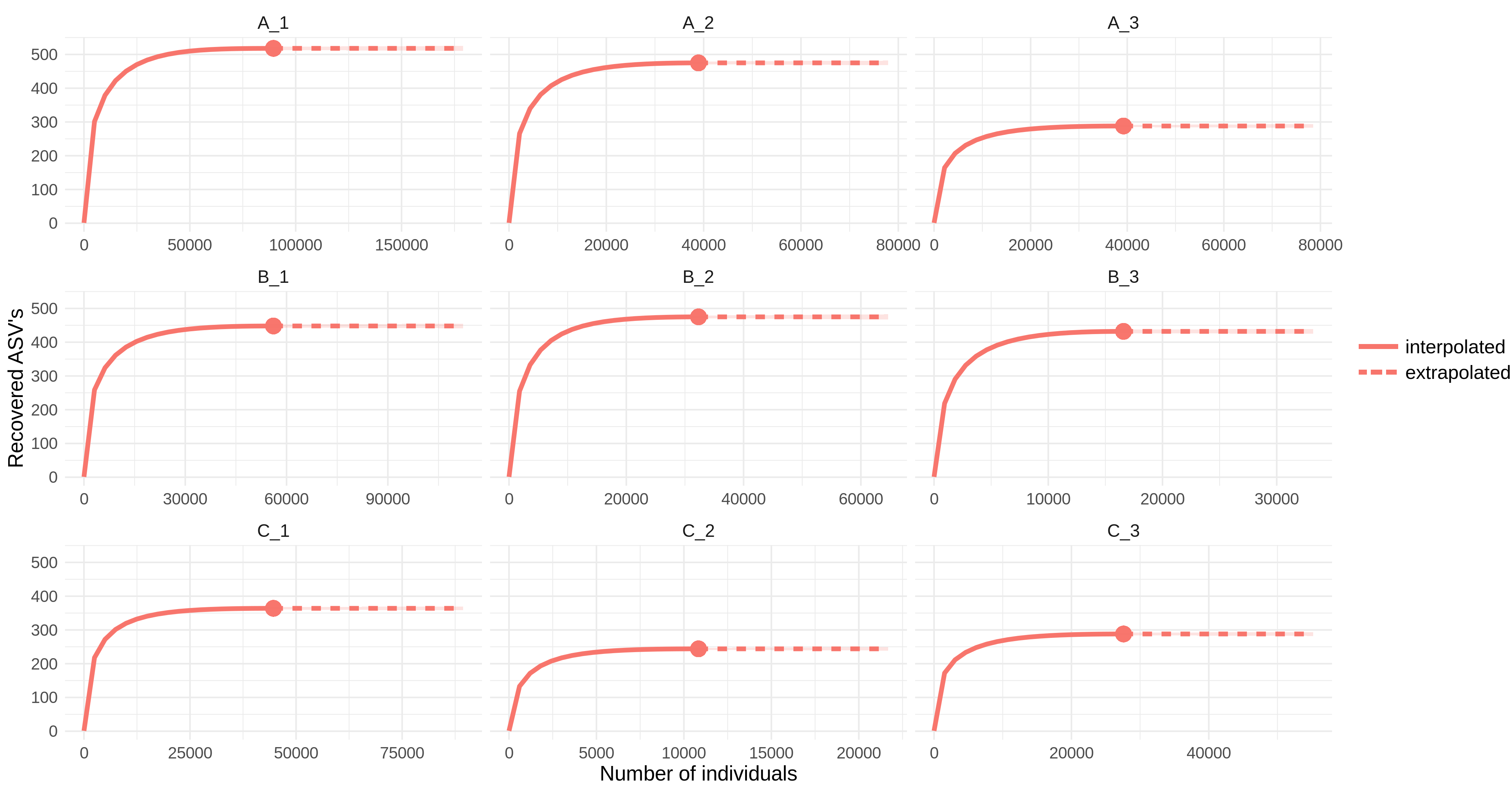

Supplement: Supplemental Information 4 — Accumulation curves of community richness estimates at different sampling sites (A, B, C) and subsamples (1, 2, 3). [file peerj-09-11956-s004.tiff]

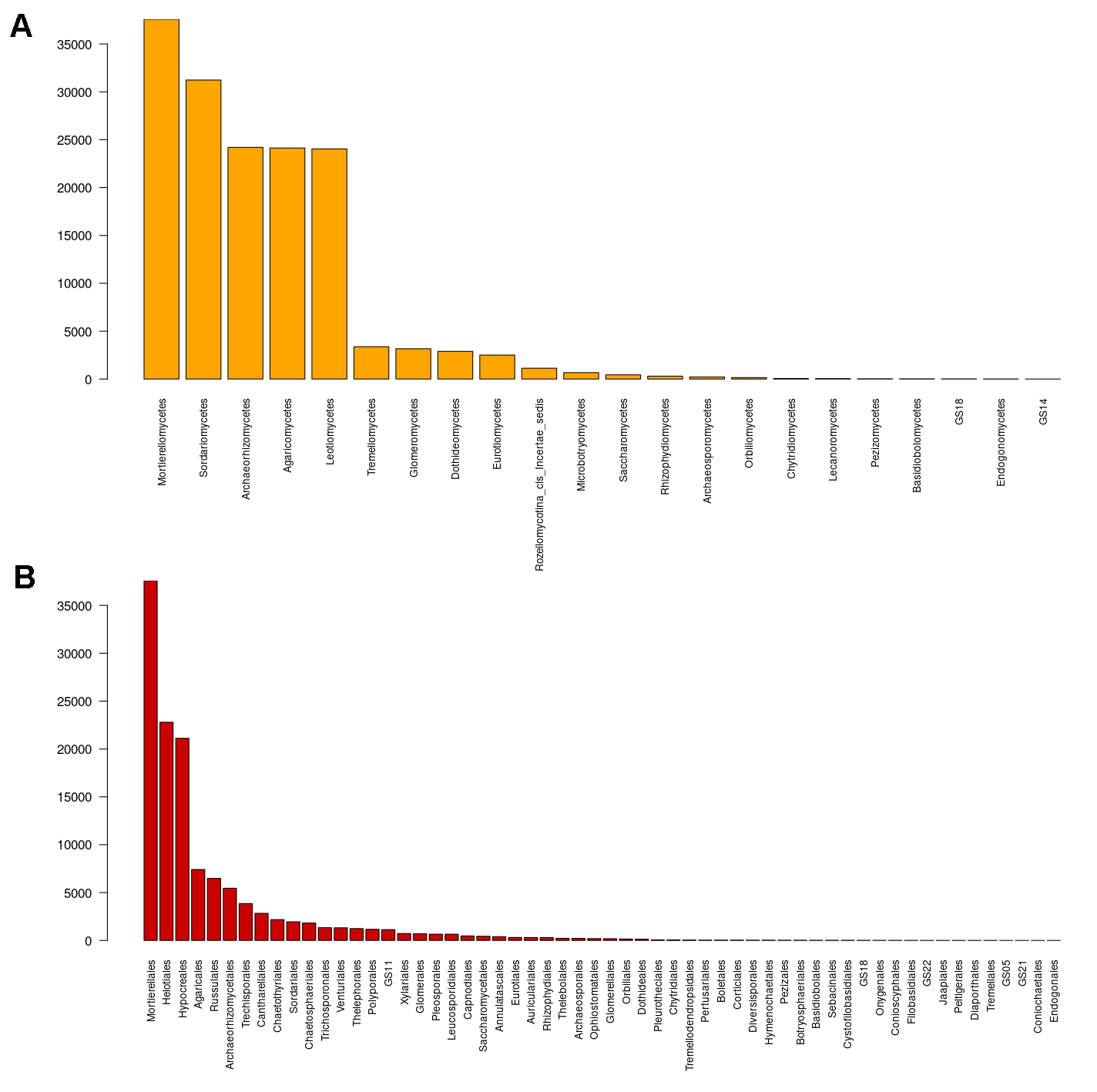

Supplement: Supplemental Information 5 [file peerj-09-11956-s005.png]
